# Design of all-optical, hot-electron current-direction-switching device based on geometrical asymmetry

**DOI:** 10.1038/srep21470

**Published:** 2016-02-18

**Authors:** Chathurangi S. Kumarasinghe, Malin Premaratne, Sarath D. Gunapala, Govind P. Agrawal

**Affiliations:** 1 Advanced Computing and Simulation Laboratory (A_*χ*_L), Department of Electrical and Computer Systems Engineering, Monash University, Clayton, Victoria 3800, Australia; 2 Jet Propulsion Laboratory, California Institute of Technology, Pasadena, CA 91109, USA; 3 The Institute of Optics, University of Rochester, Rochester, New York 14627, USA

## Abstract

We propose a nano-scale current-direction-switching device(CDSD) that operates based on the novel phenomenon of geometrical asymmetry between two hot-electron generating plasmonic nanostructures. The proposed device is easy to fabricate and economical to develop compared to most other existing designs. It also has the ability to function without external wiring in nano or molecular circuitry since it is powered and controlled optically. We consider a such CDSD made of two dissimilar nanorods separated by a thin but finite potential barrier and theoretically derive the frequency-dependent electron/current flow rate. Our analysis takes in to account the quantum dynamics of electrons inside the nanorods under a periodic optical perturbation that are confined by nanorod boundaries, modelled as finite cylindrical potential wells. The influence of design parameters, such as geometric difference between the two nanorods, their volumes and the barrier width on quality parameters such as frequency-sensitivity of the current flow direction, magnitude of the current flow, positive to negative current ratio, and the energy conversion efficiency is discussed by considering a device made of Ag/TiO_2_/Ag. Theoretical insight and design guidelines presented here are useful for customizing our proposed CDSD for applications such as self-powered logic gates, power supplies, and sensors.

Highly enhanced electric fields produced by localized surface plasmons inside plasmonic nano-particles can generate a high-energy non-equilibrium electron gas^1^,^2^ referred to as hot electrons. A fraction of these hot electrons often have sufficient momentum to cross the potential barrier at the nano-particle boundary and enter neighboring materials^3^,^4^. This is a fundamental process that has been found useful for many applications such as photovoltiacs^5^,^6^, photocatalysis^3^,^7^,^8^, nano-scale imaging^9^ and photodetection^10^. In this paper we exploit this process for designing a novel optically controlled, current-direction-switching device.

The number and the energy distribution of hot electrons generated inside a nano-particle depends strongly on its shape and size^1^,^2^, in addition to the nature of its composition, excitation power, excitation frequency, and dielectric properties of the surrounding medium^11^,^12^. Therefore, when two nano-particles that are dissimilar in shape or size are optically excited under similar conditions, one nano-particle can be expected to produce more higher energy electrons than the other due to this geometrical asymmetry. If they are separated by a finite potential barrier, a net flow of electrons (i.e., a net photo-current) from one particle to the other can be expected. Moreover, the direction and the magnitude of this current would depend on the excitation frequency. This process will leave one nano-particle positively charged and the other negatively charged. When an electron donor/receptor mechanism, such as an external circuit connecting both nano-particles, is available to transport the transferred holes/electrons and to maintain the charge neutrality, a continuous net electron flow can be expected. Clearly, this process can be exploited to develop a novel optically controllable nano-scale circuit element, capable of absorbing energy from an optical field to drive a current in the connected circuit, with the direction of the current switched via the applied field frequency. Moreover, the ability to control the internal electric field enhancement and the quantum-mechanical properties of nano-particles via their composition, shape and dimensions^2^,^13^ gives one the flexibility for tailoring the switching frequencies of such devices. Owing to this behaviour, we refer to such a devices as the current-direction-switching-device (CDSD) in this paper. Such nanoscale, optically controlled, power supply devices will allow designing self-powered logic-gates for energy/information processing, bio-sensors, optical sensors and power supply units, leading to nano and molecular circuits that can function without external wiring.

Similar CDSDs based on semiconductor-semiconductor or semiconductor-metal structures^14^,^15^,^16^ and molecules^17^,^18^,^19^,^20^ have been reported in literature. However, in almost all cases they are based on fluid solutions and consist of subtle molecules or semiconductors, acting as light harvesting media. Their operation is mainly based on difference in material propertied rather than difference in geometry, requiring multiple complex materials for construction. To our knowledge, the possibility of switching a plasmon-induced photo-current direction based on the geometrical asymmetry of nano-particles has not been discussed so far in the published literature. Such systems can offer higher mechanical and chemical stability than the existing fluid-based molecular systems. Furthermore, the use of metallic nano-particles as a light-harvesting medium is more desirable compared to bulk-metals, semiconductors or molecules, since plasmon-enhanced electric fields in metallic nano-particles can generate hot electrons more efficiently than the direct electron excitation mechanism used in other media. However, molecular-scale components have the potential for higher packing densities enabling highly miniaturized electronic circuits. Therefore, a hybrid approach involving a combination of molecular components with nano-scale power supply may be better suited in practice for developing electronic circuits.

It has been shown experimentally in the context of light-harvesting applications that a sustainable electron flow in a circuit based entirely on optically excited electrons generated from a metallic nano-structure is possible^3^,^4^,^21^. For example, an efficient solar water-splitting system with light as the only energy input has been demonstrated^3^. Its operation is based on Ag nanorods with TiO_2_ caps that are submerged in water. A portion of electrons generated in an Ag nanorod passes through TiO_2_ and is eventually transferred to ionized hydrogen in water, generating H_2_ and leaving the nanorod positively charged. Electrons from the oxygen ions enter the Ag nanorod to resupply it with electrons, ultimately creating an electric circuit for the electron flow. Also, the ability to extract and inject hot electrons from plasmonic nano-particles to molecular electronic devices, has been experimentally demonstrated in a system consisting of Au nano-particles linked to thiophenylethynyl-terminated porphyrin molecules^6^ where a hot-electron based current was clearly observed in the molecular structure. Such observations confirm the possibility of generating a continuous photo-current from plasmonic nano-particles that can be injected to a molecular- or nano-structure^22^ acting as the external circuit.

The efficiency of hot-electron generation and injection depends significantly on the frequency-dependant enhancement of the electric field inside a nano-particle, which is essentially decided by its shape. Rod-like or ellipsoidal shapes supporting a longitudinal plasmon resonance have been proven to be more efficient than other basic shapes such as nanospheres, nanocubes or nanopallets^1^,^2^. Furthermore, the direction of the applied electric field relative to the nano-particle-barrier interface has a high influence on the generation of hot electrons with correct momentum orientation for injection over the barrier. Therefore, when designing self-powered hot-electron based CDSDs, a ‘rod-like’ shape is most suitable for the plasmonic nano-particle together with an optical field polarized along its longitudinal direction.

The non-equilibrium excited electrons relax into lower energy levels by emitting photons, distributing their energy to other electrons (in several hundred femtoseconds), and transferring energy to the lattice within a few picoseconds^23^,^24^,^25^. For this reason, the injection of hot-electrons over an energy barrier should happen before electrons lose energy through relaxation processes. On the other hand, such fast relaxation allows the hot-electron-based devices to have switching speeds approaching terahertz frequencies^26^.

In this work, we have designed an all-optical, nano-scale, hot-electron CDSD by using two dissimilar metallic nanorods, separated by a thin potential barrier in the middle. The proposed structure require only two materials that can be found in abundance: a plasmonic metal such as gold or silver and a wide-band-gap semiconductor such as TiO_2_. Such a device is easy to fabricate and is also economical compared to most other existing designs found in literature, which are made of complex molecules or multiple semiconductor-insulator layers. A continuous photo-current with the direction controllable via the operating frequency, can be injected from this CDSD to a molecular or a nano-structures acting as the external circuit. Moreover, since this CDSD is powered and controlled optically, it can be used to build all-optical nano/molecular circuits.

Typically, Fowler’s theory is used to quantify the rate of electron injection from a metal to another material in contact^27^. This theory assumes an isotropic distribution of electron momentum orientations inside the metal, which is not the case for small nano-particles with dimensions less than the electron mean free path^28^. At energies close to the Fermi energy, the electron mean free path for metals used for hot electron generation such as silver and gold have reported values of 50 nm and 40 nm, respectively^29^,^30^. Therefore, for nano-structures such as the ones used in our design, a quantum-mechanical model incorporating the shape, size and composition specific effects of nonuniform electron energy distribution has to be developed. We model the behaviour of electrons inside the plasmonic nano-particles of our proposed CDSD by using the wave function for an electron confined to a finite potential well and consider the effect of the internal electric field as a periodic perturbation causing electronic transitions among energy levels. Similar models have been successfully applied for modeling hot-electron generation in isolated geometries such as nanorods, nano-pallets, nano-cubes^1^,^2^,^31^, and nanospheres^32^. These results have been successfully matched with those obtained with the density functional theory (DFT), an high-accuracy ab-initio approach that includes quantum-mechanical many-body effects but is limited to smaller nano-particles (dimensions <5 nm) owing to its excessively high computational costs.

## Theoretical Formulation

**Figure 1:**
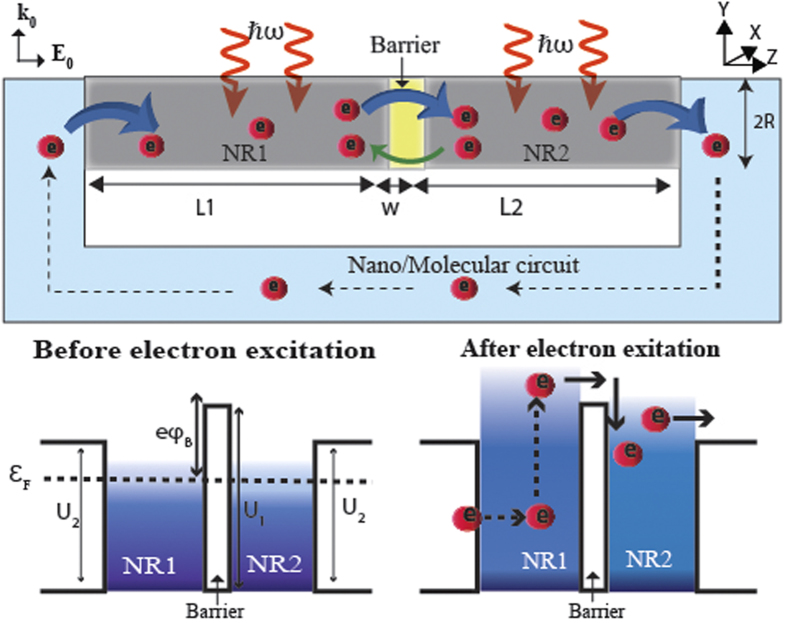
Schematic showing generation of hot electrons inside two metallic nanorods (NR1 and NR2) and their injection from one nanorod to the other over a potential barrier. The incident light is propagating in the direction of the wave vector **k**_**0**_ with its electric field **E**_**0**_ oriented along the length of nanorods. The bottom part shows the energy-band diagrams before and after the optical excitation.

As shown in Fig. 1, we consider two metallic nanorods of different lengths *L*_1_ and *L*_2_ but the same radius *R*. Both nanorods are made of the same material but are separated by a thin potential barrier of width *w*. The barrier height is given by 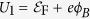, where 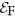 is the Fermi level of the metallic nanorods and *eϕ*_*B*_ is the potential difference between 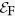 and the conduction band of the barrier material (see the bottom part of Fig. 1). We assume that our structure is connected to an external circuit that allows flow of electrons. As seen in the figure, *U*_2_ is the potential-barrier between the external circuit and the nano-particle.

When the nanostructure shown in Fig. 1 is optically exited with an electric field oriented along the nanorod length, hot electrons are generated in both nanorods. A fraction of these electrons attain sufficiently high energy with desired momentum orientation to cross the potential-barrier in the center and enter the neighboring nanorod. The electric field enhancement in two nanorods is different owing to the difference in their lengths. Also, the electron wave-functions and energy levels in each are different due to differences in electron confinement. Consequently, the energy distribution of hot electrons generated and the number of electrons that cross the potential-barrier turn out to be unequal for each nanorod. This results in a net electron flow across the barrier from one nanorod to the other, generating a net electron flow in the connected external circuit. Since, which nanorod generates more high-energy electrons is decided by the excitation frequency, the direction of the net current flow over the barrier is controllable via the excitation frequency. In this paper, we refer to current flow from NR_1_ to NR_2_ as being positive (and from NR_2_ to NR_1_ as being negative).

In the following, we first outline the procedure for deriving a general expression for the net electron flow over the potential-barrier separating two nano-particles. Then we apply this expression to the nanostructure in Fig. 1 by calculating the wave functions and electron energy levels for the nanorods in the presence of an internal electric field while also paying attention to the specific nature of their boundaries.

### General expression for the rate of electron flow

As discussed in the Methods section, the time-dependent transition probability for an electron from an initial state *i* to a final state *f* under an external perturbation *V*′(**r**, *t*) can be written as


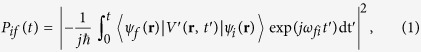


were 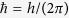, *h* is the Plank’s constant, *t* is the time measured from the start of the perturbation, 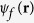 and 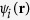 represent electron’s wave functions in the final and the initial states, and *ω*_*fi*_ is the energy difference between these two states. The rate of electron excitation 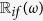 can be obtained by taking the derivative of *P*_*if*_(*t*) with respect to time. For a sinusoidal perturbation at frequency *ω*, i.e. 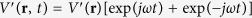, this rate is found to be


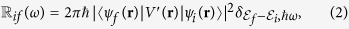


where 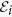 and 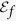 are eigen-energies of the initial and the final states of the transition and 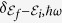 is the Dirac delta function.

In deriving Eq. (2) from Eq. (1) we have assumed that the duration of the perturbation, *t* is much greater than the electron relaxation time associated with electron-electron collisions^33^,^34^. This allows us to use the approximation


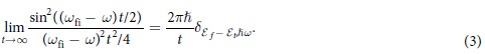


Electrons that are excited to high energy states either surmount the potential barrier and reach the other nano-particle or reflect off the barrier due to scattering events. The probability of an electron with energy 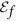 crossing the barrier can be written as^35^,^36^,


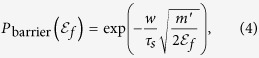


where *m*′ is electron’s effective mass and *τ*_*s*_ is electron’s scattering time in the barrier. As expected, higher energy electrons have a larger probability of crossing the barrier. In nanometer-sized semiconductor particles, the magnitude of band bending is negligibly small^37^,^38^ and therefore safely discarded in our derivations.

By summing 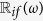 over all possible initial and final electron states and accounting for the barrier loss and electron availability, the transfer rate of hot electrons from one nano-particle to the other nano-particle can be obtained as


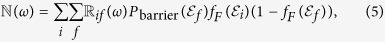


with 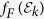 indicating the Fermi distribution associated with an electron of energy 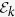. The multiplication factors 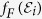 and 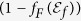 account for the the probability of finding an electron in the initial state *i* and the probability of finding the final state *f* empty during a transition. This equation has been multiplied by a factor of 2 to account for electron’s spin and by a factor of 1/2 to account for the probability of the excited electron having its momentum towards the barrier (instead of the opposite direction). However, the two factors simply cancel each other.

If 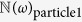 and 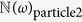 represent the hot-electron transfer rates from each nano-particle to the other particle, the general expression for the electron flow rate between the CDSD and the external circuit can be written as


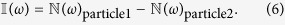


This equation can be used for any nano-particle shape after substituting relevant perturbing potentials along with electron wave functions and energy levels. In the following sections we apply it for the CDSD described in Fig. 1 and consisting of two nanorods.

### Perturbing potential inside the nanorods

The time-dependent external electric field 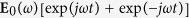 of the incident light is assumed to be linearly polarized along the longitudinal axis of nanorods since longitudinal modes of electrons show higher field enhancement than the transverse modes in rod-like shapes^39^. We write the amplitude of the internal electric field in the form **E**(*ω*) = *γ*(*ω*)**E**_0_(*ω*), where *γ*(*ω*) is the the enhancement factor induced by localized surface plasmons. This factor depends strongly on the shape of the nano-particle and its orientation with respect to the applied field, in addition to the dielectric properties of the nano-particle and the surrounding medium^40^. Since it is known that the plasmonic behaviour of a rod-like shape is quite close an ellipsoid when its aspect ratio (length/radius) is high, we assume that the internal electric field is oriented parallel to the external field^41^,^42^. The enhancement factor then takes the form^1^,


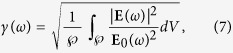


where 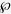 is the volume of the nano-particle.

The perturbing potential can now be written considering 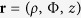 in cylindrical coordinated as


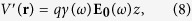


where *q* is the charge of an electron. By substituting Eq. (8) in Eq. (2) we obtain the electron transition rate as


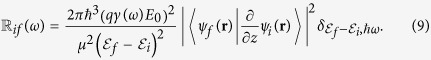


Here, we have used the quantum mechanical relationship between the position operator **r** and the momentum operator 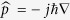 in the form^43^,


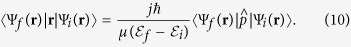


### Energy eigenstates of an electron inside a nanorod

Assuming that conduction electrons in a metallic nanorod act as free particles in a cylindrical potential well and the nanorod boundary acts as an infinite potential-barrier except in the longitudinal direction, the Schrödinger equation for a single electron can be written in cylindrical coordinates as


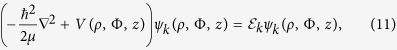


with the confining potential 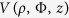 given by


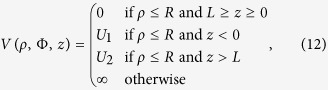


where *L* is the length of the nanorod under consideration. As shown in Fig. 1, *U*_1_ and *U*_2_ denote the heights of the potential barriers on two sides of a nanorod. Owing to the cylindrical symmetry, we can employ the method of separation of variables and write the wave function in the form


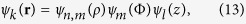


where the integers 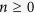, 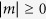 and *l* ≥ 0 are quantum numbers corresponding to a particular electron state *k*. Here, 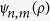, 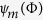, and 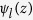 represent the radial, azimuthal and longitudinal components of the wave function respectively. The electron energy of state *k* can be written as,


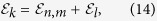


with 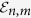 representing the radial and azimuthal components and 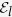 representing the longitudinal component.

After applying the orthonormal property of the radial and azimuthal components of the wave function we can simplify Eq. (9) as


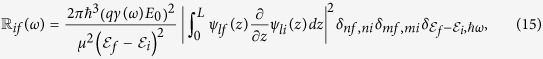


where the quantum numbers *mi*, *ni* and *li* coorespond to state *i* and *mf*, *nf* and *lf* correspond to state *f*. It is clear in Eq. (15) that the conditions *mi* = *mf* and *ni* = *nf* are required for 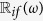 to be non-zero, and we need to consider in our analysis only those transitions that change the longitudinal quantum number. Physically, this condition is imposed because the applied electric field is along the longitudinal axis of the nanorod, and the internal electric field is in the same direction as the applied field.

The electron is confined by infinite boundaries in radial and azimuthal directions according to Eq. (12) and therefore the quantized electron energies 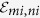 and 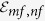, required for further calculations can be easily found as shown in the Methods section. However, finding the longitudinal components 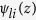, 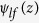, 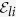 and 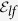 is more complicated owing to the finite nature of longitudinal boundaries. To find these we assume that prior to excitation electrons reside in an energy state below the Fermi level 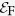, which is less than both *U*_1_ and *U*_2_, and all final energy states of interest have longitudinal energies larger than *U*_1_ and *U*_2_. As shown in the Methods sections, two sets of solutions needs to be found for Eq. (11) for these two situations^33^. From these solutions it is clear that 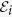 is quantised. However, 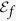 is not quantized because 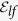 is continuous as it is greater than both *U*_1_ and *U*_2_ i.e., in the final state the electron is unconfined in the longitudinal direction.

### Electron flow over the barrier as a function of frequency

After substituting the initial and final wave functions derived in the Methods section (Eqs (30) and (36)) into Eq. (15), we obtain the transition rate in the form


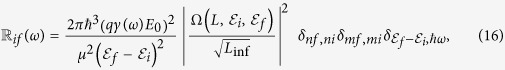


where,


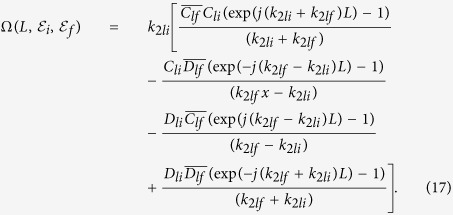


In the Methods section it is described how to find the values of *C*_*li*_, 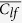, *D*_*li*_, 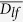, *k*_2*li*_ and *k*_2*lf*_ in terms of *L*, 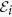 and 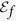. The variable *L*_inf_ is a fictitious length of confinement that is introduced for the ease of final eigen states derivation, which is removed at a later stage.

Because of the existence of a continuum of final states we can write the summation over discrete final states in Eq. (5) as an integration over energy 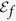
43. The result is


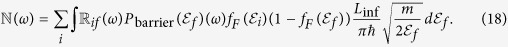


We can safely approximate the probabilities of finding the state 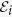 occupied and state 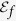 empty given by 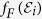 and 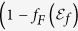 as unity because 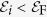 and 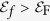 in all situations of interest. By substituting Eq. (16) in this equation, we finally obtain the bound-to-continuum electron transfer rate for a nanorod:


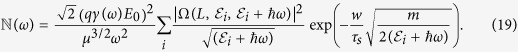


The electrons will initially occupy a state below the Fermi level. After the transition, in order to be injected to the barrier, electrons should have longitudinal energy greater than the barrier indicated by 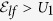. Therefore considering a linear single-photon absorption, the limits of the summation for the quantum number *i* = {*mi*, *ni*, *li*} can be given as,


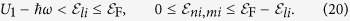


The most important feature of Eq. (19) is that the electron transfer rate depends on the length of the nanorod and is different for the two nanorods in Fig. 1 because of their different lengths. We can calculate the net current flowing through the circuit by using


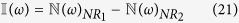


where the subscripts indicate the two nanorods of different lengths. We present the numerical results based on Eqs (19) and (21) in the next section.

## Results and Discussion

We consider a CDSD whose two nanorods made of silver are separated by a thin TiO_2_ barrier. TiO_2_ is selected as the barrier material because it has a wide-band-gap of 3.3 eV and it does not absorb radiation below this band-gap value. Therefore, in the frequency range 1.5 to 3.3 eV, we can neglect any contribution from excited electrons in TiO_2_ to the net electron flow. It is important that the barrier material does not generate excited electrons contributing to the electron flow because we do not have control over the direction of such electrons. Also it could create more resistance for the electrons crossing the barrier. Silver is selected as the plasmonic metal for the nanorods due to its strong interaction with light.

The parameter *U*_1_ can be found as 
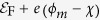
, where *ϕ*_*m*_ is the work function of Ag (in the range 4.26–4.72 eV)^44^ and *χ* is the electron affinity of TiO_2_ films (about 3.9 eV)^45^,^46^,^47^. This gives *U*_1_ = 0.8 eV for our CDSD, and this barrier height is adjustable by changing the materials used. For example by using Ag with SiC poly-types for which the electron affinity can be varied between 2.33–4.00 eV^48^,^49^, barrier height can be adjusted between 0.70–1.57 eV. For all calculations, we assume the potential-barrier *U*_2_ between the nanorods and the external circuit is 0.5 eV.

**Figure 2:**
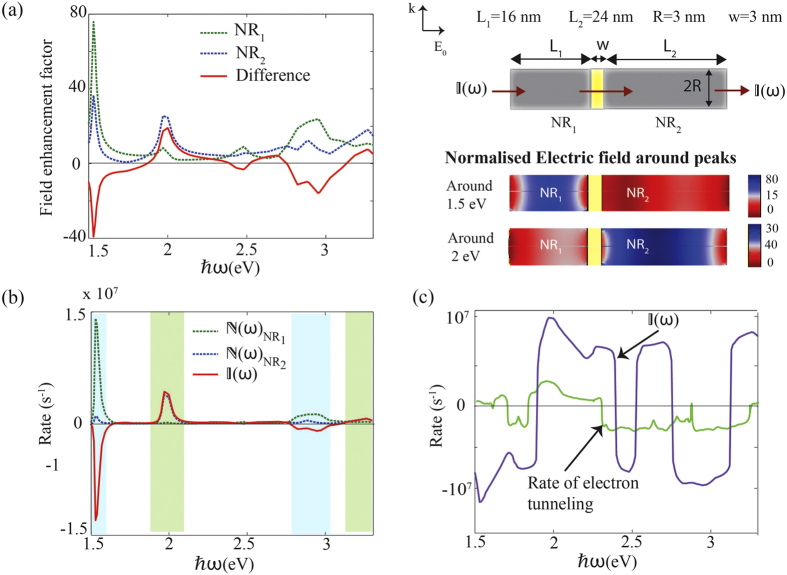
Hot-electron switching behaviour in two nanorods. (**a**) Electric-field enhancement factors (*γ*(*ω*)) inside NR_1_ and NR_2_ and their difference. The Ag/TiO_2_/Ag composite structure and its design parameters along with the normalised electric field (**E**(*ω*)/**E**_0_(*ω*)) in a cross section of the device around the peaks 1.5 eV and 2 eV are shown on right. (**b**) Injection rates of hot electrons from NR_1_ and NR_2_ and the net current flow through the CDSD. The blue and green bands indicate the direction of current flow that can be switched by changing photon energy. (**c**) Comparison of net current through the CDSD and the tunnelling current through the barrier (on a log scale).

Figure 2 shows the electric field distributions, electric field enhancement factors and the resulting hot-electron injection rates for the two nanorods along with their differences. It can be seen from Fig. 2(a) that electric field enhancement factors are different in the two nanorods, and this difference is frequency dependent, showing negative and positive peaks at certain frequencies. For instance, NR_1_ is at resonance around 1.5 eV while NR_2_ is not. Therefore, as shown in the cross section view, the electric field intensity inside NR_1_ in general is much stronger than in NR_2_ at this frequency, generating a field asymmetry inside the CDSD. This contributes to unequal hot electron generation in nanorods and as a result a net current flow from NR_1_ to NR_2_. However the field asymmetry is reversed around 2 eV, where the electric field inside NR_2_ in general appears to be much stronger than in NR_2_ due to resonance, contributing to a net electron flow from NR_1_ to NR_2_. Figure 2(b) illustrates how the current flow over the barrier from each nanorod is variable with the frequency. When 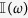 is positive, the current flow is from NR_1_ to NR_2_; the direction reverses when 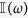 is negative. When comparing Fig. 2(a,b), it is apparent that, as a general trend, peak values in 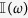 correspond to the peaks in the enhancement factor difference. Figure 2(c) shows 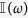 on a logarithmic scale and shows more clearly the nature of frequency dependant direction switching. The main thing to note is that both the magnitude and the direction of net electron flow can be easily controlled by varying the excitation frequency.

Electrons with longitudinal energy less then the barrier height can still pass through the barrier via tunnelling. The tunnelling transmission coefficient for the barrier can be calculated using^33^,


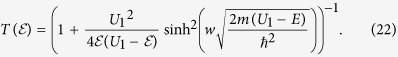


The tunneling-aided electron transmission rate can be calculated by summing up all energy states below *U*_1_ multiplied by 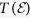. However, it can be seen from Fig. 2(c) that for the electric field intensity (3.6 × 10^7^ Wm^−2^) and the barrier width of 3 nm that we have considered here, tunnelling current is negligible compared to 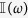. Experimental studies also suggest that tunneling of electrons between plasmonic nano-particles is negligible for junction widths larger than 0.5 nm^50^ under moderate electric field intensities used here.

**Figure 3:**
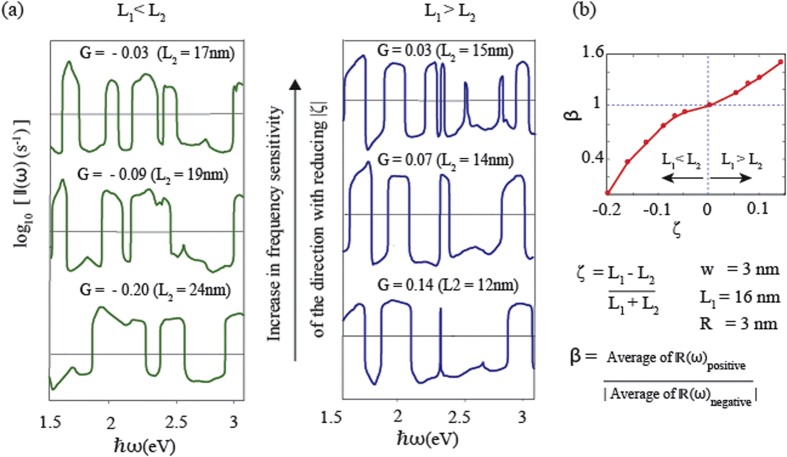
Effect of relative length difference. All parameters are the same as in Fig. 2 except *L*_2_ is varied from 12 nm to 22 nm keeping *L*_1_ and all other parameters constant. (**a**) 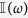 as a function of photon energy on a log scale. (**b**) *β* as a function of *ζ*.

In Fig. 3, we vary the length of one nanorod while keeping length of the other nanorod constant to study the effects of geometrical asymmetry on 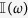. We introduce the parameter, 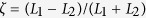, representing the relative length difference of two nanorods. It can be seen from Fig. 3(a) that as the value of *L*_2_ gets closer to *L*_1_ so that the absolute value of *ζ* becomes small, the oscillatory nature of 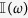 becomes more pronounced, making the direction of the flow highly sensitive to the frequency. We define another parameter *β* to represents the ratio between average values of the current in the positive and negative directions. In designing CDSDs this ratio is an important parameter and its ideal value is close to unity. It can be seen from Fig. 3(b) as the absolute value of *ζ* becomes large, *β* moves away from unity, indicating an increased difference between magnitudes of positive and negative currents.

**Figure 4:**
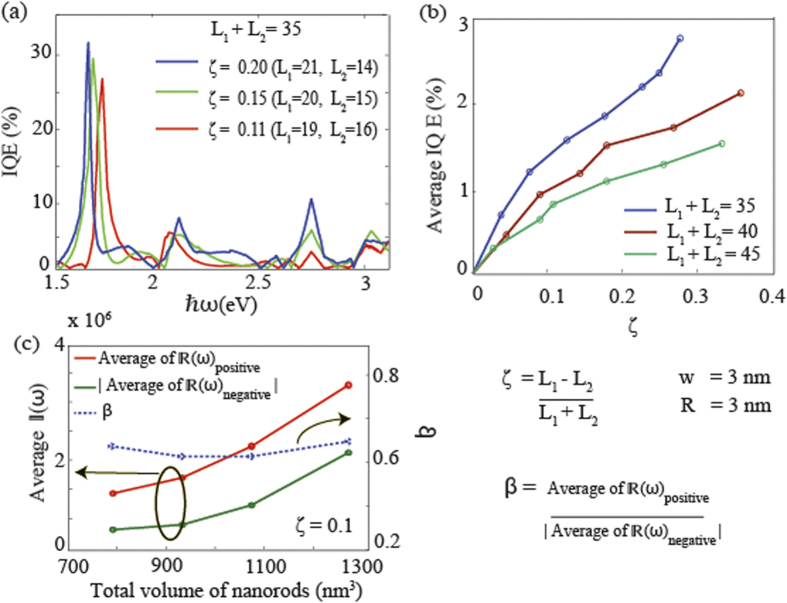
Same as in Fig. 2 except *L*_1_ and *L*_2_ are varied keeping the value *L*_1_ + *L*_2_ and other parameters constant. (**a**) 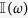 in log scale for different *L*_1_ and *L*_2_ combinations. (**b**) Quantum efficiency for each case as a function of frequency. (**c**) Averaged quantum efficiency for each case against the ratio *L*_1_/*L*_2_.

Figure 4 shows the influence of relative nanorod length difference (*ζ*) and overall device volume on the energy conversion efficiency and the magnitudes of positive and negative current flows. The internal quantum efficiency (IQE) is a measure of energy conversion efficiency of a system and can be used as a figure of merit for comparison among different energy converting systems. For this nano-structure, IQE can be defined as the ratio of absorbed photons to injected net electrons over the barrier (see Methods). Figure 4(a) shows IQE for devices having different combination of *L*_1_ and *L*_2_ (i.e. different *ζ* values), while keeping total length *L*_1_ + *L*_2_ constant. It can be seen that the IQE exhibits an oscillatory behavior. However, note that the peak quantum efficiency can exceed 30% for *ζ* = 0.2 at a certain excitation frequency. Figure 4(b) shows the averaged IQE over the total range of photon energies (1.5–3.2 eV) for different device volumes and *ζ* values. An increase in the value of *ζ* can increase the averaged IQE, indicating that a higher geometric difference can lead to higher efficiency. Further, for a certain *ζ* value, increasing the overall device volume can result in reduced averaged IQE. At the same time, since the number of conduction electrons increases with increasing volume, the magnitude of current flow (averaged over the spectrum), both positive and negative, increases with the volume as seen in Fig. 4(c). However, the the ratio between average positive to negative electron flow (*β*) remains almost constant despite of the change in volume, indicating it is mainly dependant on the geometrical deference indicated by *ζ*.

**Figure 5:**
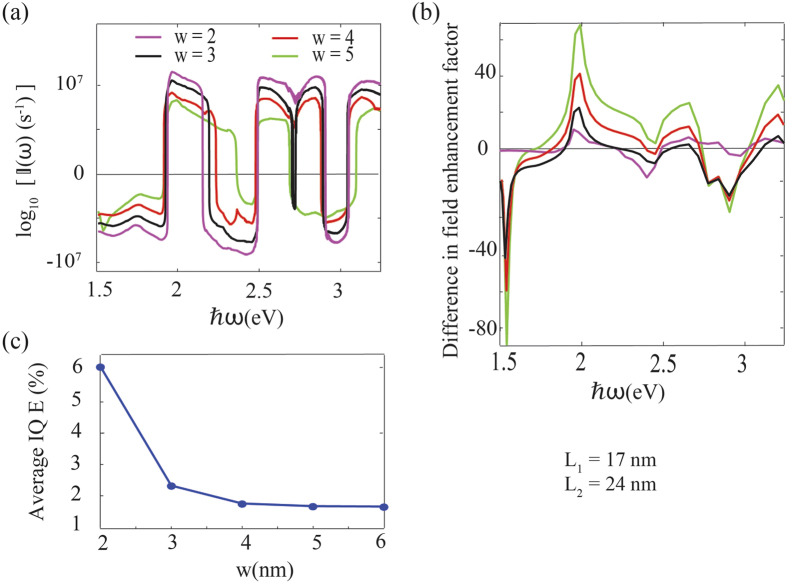
Same as in Fig. 2 except that the barrier width is varied while keeping other parameters constant. (**a**) 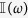 for different *w*. (**b**) Difference in the electric field enhancement factor. (**c**) Averaged quantum efficiency for each case as a function of frequency.

In Fig. 5, the barrier width is varied while keeping all the other factors constant. When the separation between nanorods is high, as seen in In Fig. 5(b), the difference between their electric field enhancements will also increase due to reduced electric field coupling between nanorods, which can contribute positively for improving 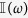. On the other hand, the electron energy loss while crossing the barrier will increase with increasing *w*, which can have a negative effect on 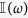. However, as seen in Fig. 5(a,c), the overall effect is a reduction in the net electron flow and the averaged IQE.

In our derivations, the duration of the electron excitation under the optical field is considered to be much greater the electron relaxation time associated with electron-electron collisions. Under such conditions, electrons achieve local equilibrium within the duration of the laser pulse and the metal nanoparticle thermodynamic state can thus be described by one temperature. Therefore the current injection rate from the CDSD to the external circuit is found to be independent of time during the process. For Ag, this electron relaxation time in literature is typically around 350 fs^24^,^51^,^52^.

If the pulse duration is shorter than this electron relaxation time, the electron flow rate between the CDSD and the external circuit during and after the optical perturbation need to be studied in a high resolution femtosecond or picoseconds time scale emphasising non-equilibrium effects which is outside the scopes of this research. Under a picosecond pulse, before the end of the pulse, the electron gas achieves internal thermalization vial electron-electron collisions, whilst the electron-lattice system is still far from equilibrium. Under these conditions the classical two-temperature model^24^,^51^,^53^ is used to model the time evolution of electron excitation rate due to energy exchange with the lattice. This energy exchange rate is proportional to the lattice-electron temperature difference and the electron-phonon coupling constant of the nanoparticle^24^,^53^,^54^. Under a femtosecond laser pulse, electron-electron collisions are not fast enough to thermalize the electron gas during the laser pulse, therefore the non-equilibrium conditions are maintained up to some hundreds of femtoseconds. Under this condition, the Boltzmann equation should be solved for both electrons and phonons, considering the Fermi-Dirac and Bose-Einstein statistics, respectively to calculate the energy distributions for the two systems.

A challenge in fabrication of any nano structure is to control the particle sizes, shapes and morphologies. Irregular shapes and sizes due to fabrication imperfections may change the electric field enhancement and the electron energy structure of the CDSD from expected values. The geometry of the tips of the rods have a significant impact on field enhancement, especially in smaller rods where the tips comprise a larger portion of the overall rod^55^. Sharper tips generate higher field enhancement and the lightning-rod effect can be seen in extreme cases^56^.

Irregular surfaces with pits and bumps can be approximated roughly as an array small spherical or spheroidal particles, each having its own resonance frequency sitting on a flat surface^57^. If there are only few such irregularities, random peaks can be observed in the field enhancement spectra. If the surface is highly irregular containing pits and bumps of different sizes and shapes, it can broaden the peaks of the field enhancement spectrum due to combined effects, resulting in reduced frequency sensitivity for current direction switching.

The magnitude of the output is highly sensitive to the barrier width as discussed in the previous section. A few nanometre difference in the barrier thickness can cause a few orders of magnitude change in the output current. The current switching frequencies and internal quantum efficiencies can be considered as moderately sensitive to device dimensions.

## Design Guidelines and Conclusion

We have proposed a composite nanostructure that is capable of generating a current flow in a connected circuit when light is incident on it. Moreover, both the direction and the magnitude of current is controllable via the frequency of the incident light. The device operation is based on the concept of hot-electron injection from two geometrically dissimilar nano-particles over a common barrier. We have derived the rate of electron flow in such a structure, made of two nanorods, by using the single-electron wave function and the corresponding eigen energy states of an electron inside each nanorod under a periodic perturbation. The electric field enhancement inside the structure, which has a high influence on the hot-electron generation rate was derived numerically.

We used our theoretical model to study how the current flow in our proposed CDSD depends on various design parameters. Our results show that the magnitude and the direction of current 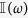 is highly controllable via the excitation frequency. Increase in the device volume can result in a decrease in the efficiency of the device and its suitability for nano/molecular scale designs. Further, if the nanorods have dimensions longer than the electron mean free path of the metal, electrons may relax before reaching the boundary, reducing the efficiency. On the other hand, the magnitude of electron flow (averaged over the spectrum) increases with the volume. Therefore, when deciding device dimensions, a compromise between the efficiency and the magnitude of current generated has to be made.

The relative lengths of the nanorods is another important parameter that effects the CDSD performance. A higher geometric difference between the two nanorods (i.e. a larger *ζ* value) can lead to a higher internal quantum efficiency but it will make the direction of the flow less sensitive to the frequency. Also a larger *ζ* will result in a higher *β* value, indicating uneven positive and negative current flows, which can be desirable or undesirable based on the application.

Decreasing the barrier width will exponentially increase the magnitude of the electron flow from the device and its internal quantum efficiency. But if the barrier width is too small, tunnelling breakdown can happen^58^, depending on the intensity of the applied electric field, leading to reduced quantum confinement and decreased controllability over the electron flow direction. Further, the difficulty in fabricating very narrow barriers must also be considered. Higher intensity electric fields can lead to an increased 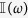 at all incident frequencies. However, the intensity of the electric field is limited in practice by the barrier width. For moderate optical intensities (~10^7^ Wm^−2^), a barrier width of about 3 nm is found to be most suitable.

A wide-band-gap semiconductor such as TiO_2_, SiC, or ZnO is desirable for constructing the barrier. The upper limit of the operating frequency is limited by the barrier band-gap since electron excitations in the barrier has to be avoided. Also the metal-barrier potential difference should be higher than the metal-external circuit potential in order to control the direction of the flow. For constructing the nanorods, strongly light absorbing plasmonic metals such as silver or gold are most suitable. The lower limit of the operating frequency is decided by the minimum energy that an electron can absorb to surmount the barrier, which is equal to the energy difference between the barrier and the Fermi level of the plasmonic metal.

When designing the proposed CDSD, Eq. (21) can be used to plot 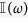 over the entire frequency rage of the incident light for different sets of *L*_1_, *L*_2_, *R* and *w* values. Then, based on the trends we have identified in this paper, the parameters, *ζ*, overall volume of the nanorods and the potential-barrier width can be varied to tune this CDSD to work for certain operating frequencies delivering required amounts of negative and positive currents to the external circuit with a desired quantum efficiency. For the dimensions we have considered in this paper, the proposed CDSD shows an average internal quantum efficiency of over 2%, with peak efficiencies approaching 30% at certain frequencies of incident light. The possibility of increasing the internal quantum efficiency by using more conductive barrier materials needs to be investigated further. Also, other combinations of particle geometries may perform better and improve the performance of such devices.

In our study the injection rate of hot electrons over the barrier is calculated assuming a smooth interface between the metal and the semiconductor. This assumption imposes the condition that the component of electron’s momentum normal to the interface should be large enough for the electron to cross the Schottky barrier. In practice, imperfections and roughness of this interface can change the direction of momentum of incident electrons and enhance the injection rate. Tunneling through the barrier is found to be negligible for the barrier thicknesses we have considered.

Coupled plasmon resonances created by plasmonic nano antennas placed in close proximity to the CDSD can generate internal fields that are enhanced by orders of magnitude, improving the efficiency of the device. The energy from these antennas can be transferred efficiently to the plasmonic nanoparticles non-radiatively through resonance energy transfer^59^,^60^ or radiatevely, depending on the distance between the antenna and the CDSD. Cascaded plasmon resonances^61^,^62^, constructed using chains of such geometrically asymmetric antennas having the same plasmon resonance frequency but significantly different volumes, have proven to provide extremely strong internal field enhancements. When such nanoparticles are coupled with each other, a multiplicative field enhancement can be observed predominantly in the smallest particle of the chain. This mechanism can be used to improve the efficiency of the proposed CDSD.

Another experimentally demonstrated method to profoundly improve the electric field enhancement in metal nanoparticles is near-field coupling with an active substrate such as Si^63^ or quantum-dot-embedded dielectrics^64^. The energy emitted by electron relaxations in the active medium can be transferred non-radiatively to the surface plasmons in the plasmonic nanoparticle. The stimulated nature of this energy transfer causes buildup of macro-scopic numbers of coherent surface plamons in the nanoparticle, increasing its internal electric field. This concept can be used to improve the electric field inside the plasmonic nanoparticles in the proposed CDSD, which then acts similar to the resonant cavity in a spaser^65^,^66^.

## Methods

### Electron excitation probability in nano-particles under an external pertubation

Prior to the optical excitation, the system is considered to be under a time independent but spatially varying potential *V*(**r**). The Schrödinger equation for the motion of an electron in this system has its usual form


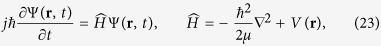


where 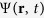 is electron’s wave function at time *t* located at a position **r**, 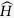 is the Hamiltonian, and *μ* is the effective mass of the electron. Since *V*(*r*) is time independent, 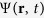 can be separated into temporal and spatial components as 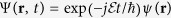. By substituting this in Eq. (23), the time independent part of the wave function satisfies the eigenvalue equation


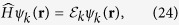


where 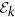 and 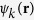 are the eigenvalues and eigenfunctions of the *k*^*th*^ solution of this equation, representing possible electron states.

When light is incident on an electron, its motion of an electron is governed by


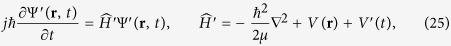


where *V*′(*t*) is the time-dependant perturbing potential causing electronic transitions between its energy states and 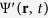 is the modified electron wave function at this stage. We can express 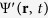 as an expansion of the eigen functions 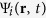 of the unperturbed, time independent system using a time dependent coefficient *c*_*i*_(*t*) as,


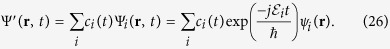


Substituting this equation in Eq. (25), multiplying the resulting expression by 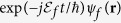, and integrating over the spatial coordinates yields,


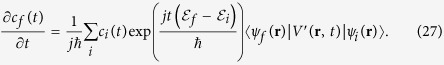


The quantity 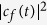 represents the time-dependent transition probability of an electron from an initial energy state *i* a final state *f*. It is given by^33^,^34^,


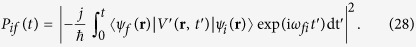


with *ω*_*fi*_ representing the energy difference between the final and initial states.

### Derivation of energy eigenstates of an electron inside a nanorod

By solving Eq. (11) for the radial and azimuthal potentials given in Eq. (12), the quantized electron energy in these directions can be easily found as^67^


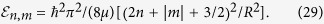


The longitudinal part of energy containing the quantum number *l* is more complicated owing to the finite nature of longitudinal boundaries.

To find the longitudinal wave functions 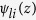 and 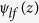 and the corresponding energy components 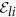 and 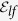, we assume that prior to excitation electrons reside in an energy state below the Fermi level 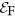, which is less than both *U*_1_ and *U*_2_, and all final energy states have energies larger than *U*_1_ and *U*_2_. Two sets of solutions needs to be found for Eq. (11) for these two situations^33^

Consider first the initial state. Since 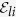 is less than *U*_1_ and *U*_2_, the wave function 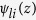 can be written in the form


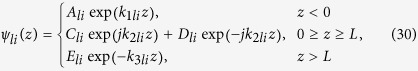


where


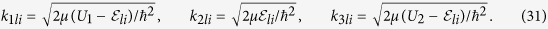


The constants *C*_*li*_, *D*_*li*_ and *E*_*li*_ can be found in terms of *A*_*li*_ considering the continuity of the wave function and its first derivative at the boundaries and are given by


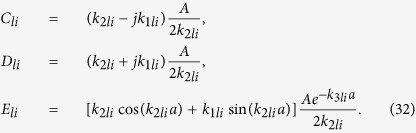


The value of *A*_*li*_ is found using the normalization 
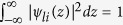
 to be


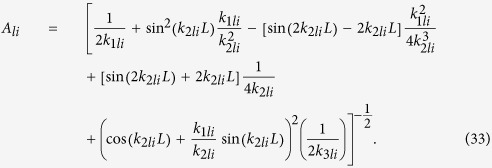


The quantized energy levels 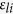 are found numerically from the eigenvalue equation


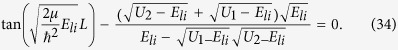


When 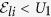 and 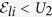, total energy of an electron in the state *i* can be written as


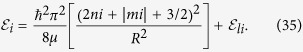


Consider now the final state. Since 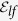 is greater than *U*_1_ and *U*_2_, 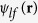 is of the form


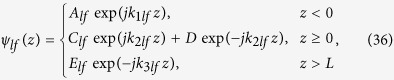


where


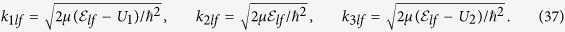


As before, the constants *C*_*lf*_, *D*_*lf*_ and *E*_*lf*_ can be found in terms of *A*_*lf*_ considering the continuity of the wave function and its derivative at the boundaries:


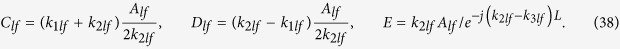


However, since there is no bounding potential, *A*_*lf*_ is not normalizable. Therefore, to find *A*_*lf*_ we introduce a fictitious length of confinement *L*_inf_ which is removed at a later stage. After applying the orthonormal conditions within this length, *A*_*lf*_ is found to be 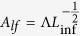 where


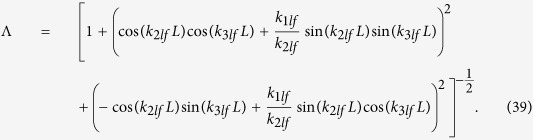


We define two new quantities,


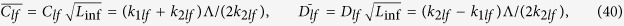


which are independent of the fictitious length of confinement *L*_inf_ and are used later. Finally, the total energy 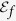 can be written as


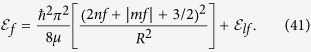


Note that 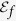 is not quantized in the *z* direction because 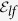 is continuous with values greater than both *U*_1_ and *U*_2_.

### Internal quantum efficiency

Internal Quantum efficiency of a CDSD can be defined as the ratio of the rate of electron injection to the external circuit 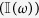 and the rate of photon absorption 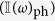 given by


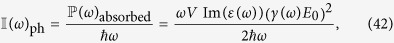


where *V* is the volume of the particle, 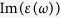 is the imaginary part of the dielectric permittivity and 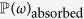 is the power absorbed.

### Material parameters

In all calculations we assume the nano-particles to be made of silver with a Fermi energy of 5.5 eV. The semiconductor is taken to be TiO_2_, creating a barrier of 0.8 eV. The mobility of electrons in TiO_2_ is taken as 1 *cm*^2^ *V*^−1^ *s*^−1 ^68,69. The complex permittivity of silver is taken from experimental data^70^ and the surrounding medium is assumed to be air with a relative permittivity of 1. The illumination intensity is taken as 3.6 × 10^7^ Wm^−2^.

## Additional Information

**How to cite this article**: Kumarasinghe, C. S. *et al.* Design of all-optical, hot-electron current-direction-switching device based on geometrical asymmetry. *Sci. Rep.* **6**, 21470; doi: 10.1038/srep21470 (2016).
